# Digital competence and psychological wellbeing in a social housing community: a repeated survey study

**DOI:** 10.1186/s12889-023-16875-2

**Published:** 2023-10-13

**Authors:** Sarah Buckingham, Gengyang Tu, Lewis Elliott, Ria Poole, Tim Walker, Emma Bland, Karyn Morrissey

**Affiliations:** 1 European Centre for Environment and Human Health, Medical School, University of Exeter, Truro, UK; 2 Centre for Geography and Environmental Science, University of Exeter, Penryn, UK; 3 Sustainability, Society and Economics Division, Department of Technology, Management and Economics, Technical University of Denmark, Lyngby, Denmark; 4 International Business School Suzhou, Xi’an Jiaotong-Liverpool University, 111 Ren’ai Rd, Suzhou, 215123 Jiangsu China

**Keywords:** Digital competence, Psychological wellbeing, Mental wellbeing, Life satisfaction, Social housing, Season

## Abstract

**Background:**

Little is known about whether digital competence is related to psychological wellbeing, with most previous research focusing on students and elderly people. There is also limited evidence on seasonal changes in psychological wellbeing, particularly in specific groups. Social housing residents are an underserved and under-researched population. The objectives of this study were to explore associations between digital competence (assessed by general technology self-efficacy) and psychological wellbeing (assessed by mental wellbeing and life satisfaction), and to explore seasonal effects, in social housing residents.

**Methods:**

A repeated survey design was used. The Happiness Pulse questionnaire with a bespoke digital module was sent via post or e-mail at four timepoints between July 2021 and July 2022 to 167 social housing residents in West Cornwall, England. There were 110 respondents in total; thirty completed all four questionnaires and 59 completed an autumn/winter and summer questionnaire. Data were analysed using descriptive and inferential methods including regression, repeated measures analysis of variance and panel analysis.

**Results:**

Significant positive associations were found between digital self-efficacy and mental wellbeing, and between digital self-efficacy and life satisfaction. However, there were no significant seasonal changes in psychological wellbeing.

**Conclusions:**

The findings extend the existing literature beyond student and elderly populations and suggest that improving digital competence is a potential pathway to improving psychological wellbeing. Surveys with larger samples and qualitative studies are needed to elucidate the mechanisms involved.

**Supplementary Information:**

The online version contains supplementary material available at 10.1186/s12889-023-16875-2.

## Background

The ubiquitous role of digital technology in everyday life has seen an increasing interest in the impact of technology on health and wellbeing. Evidence from surveys and systematic reviews strongly suggests that using technology is beneficial for mental and social wellbeing [1, 2]. It has been proposed that these benefits may be obtained via different mechanisms. These include: enabling access to online health resources [2]; improving health literacy, i.e. ability to find, understand and use health information [3]; facilitating social interactions (online and offline) [2, 4–6]; providing opportunities for professional development [6]; and enabling the development of new skills [7]. The majority of studies to date have focused on technology use (i.e. frequency and duration) rather than the wider phenomenon of digital competence. Digital competence is distinct from use of digital technologies and includes skills, knowledge, awareness, attitudes and cognition; when combined, these lead to self-efficacy [8]. Self-efficacy is an individual’s belief in their capacity to perform a behaviour (i.e. use technology) to successfully accomplish a task [9].

Studies of digital competence and psychological wellbeing have been limited with a narrow focus on specific populations. A small number of recent studies have explored the associations between digital competence and wellbeing in educational settings or with older adults. Wang and colleagues found that (survey-assessed) digital competence was associated with reduced burnout in university students; this was due to the indirect effect of digital competence on reducing cognitive load [10]. A study of university students in Poland, Lithuania, Turkey and India also found that digital competence (specifically the social and informational domains) was associated with reduced stress and burnout, in addition to higher psychological wellbeing [11]. A survey of older adults in Korea reported that smartphone use for communication (messaging and social networking) directly and indirectly impacted on life satisfaction, with digital literacy acting as a partial mediator [12]. These findings confirm the importance of differentiating between technology use and digital literacy.

Seasonal variation in physical health outcomes is well established, with excess winter deaths reported in medical journals for the last 150 years. Regarding mental health, a large clinical literature exists on seasonal variations in mood, with a higher incidence of mood and affective disorders in autumn and winter [13]. There is some evidence from the 1970s that self-reported happiness is higher in spring and summer [14], and more recently experimental wellbeing statistics for the UK have found quarterly variation in reported personal wellbeing [15]. However, the literature on the seasonality of wellbeing is more limited [16], and little is known about potential modifying and protective factors. If there is an association between digital competence and psychological wellbeing, it is plausible that digital competence may have a protective influence on seasonal related reductions in psychological wellbeing - for example, by enabling social contacts to be maintained during the shorter days of autumn and winter. To date, there is no published literature on the potential protective effects of digital competence on seasonal changes in wellbeing.

Psychological wellbeing is a complex phenomenon influenced by a range of individual (e.g. physical health, financial difficulties), community and place-based factors (e.g. social connections and neighbourhood cohesion) [17, 18]. The mental wellbeing of those living in socio-economically disadvantaged and rural areas has been identified as a public health priority [19]. These underserved communities are more likely to face hardship, which may be more pronounced in autumn and winter by increased financial burdens on households. Fuel poverty has a significant negative impact on mental health [20] and is inextricably linked with emotional wellbeing [21]. In addition, physical activity levels tend to be lower in the colder and wetter months [22], people spend less time outdoors [23], and less time socialising potentially leading to isolation [24]. As people living in socio-economically disadvantaged areas also experience greater challenges in engaging in health- and wellbeing-promoting behaviours [25, 26], seasonal impacts on wellbeing may be greater for this group. It is therefore important to understand whether seasonal variation in wellbeing exists in these communities, and to explore potential mechanisms to overcome this variation.

Social housing providers are private not-for-profit organisations that provide rental accommodation at around 50–60% of market rates for those who may be excluded from the private market due to health or economic circumstances [27]. The role of social housing providers in the UK has evolved over recent years, extending beyond simply providing accommodation to include supporting social engagement and promoting wellbeing of their residents and communities [28]. Often residing in socio-economically disadvantaged areas and reporting lower quality of life [26], social housing residents are also a group identified at high risk of digital exclusion [29, 30]. The 2021 to 2022 English Housing Survey reported that the social rented sector accounts for four million households, and that social renters are the least likely tenure to have internet access at home [31].

Smartline is a collaborative programme of research that explores the opportunities for technology to support social housing residents to live healthier and happier lives in their homes and communities [32]. The Smartline study population resides in a poor, geographically isolated rural and coastal region in Cornwall, Southwest England. Individuals in this social housing community were found to experience both social and digital exclusion; approximately 10% of residents reported feeling lonely and 21% did not have internet access [33]. Identifying modifiable factors that could improve health and wellbeing and combat social and digital exclusion in this population is therefore key.

Seeking to understand the role of digital competence (i.e. digital self-efficacy) in maintaining wellbeing in social housing residents throughout the year, we proposed three hypotheses:

### Hypothesis 1

Digital self-efficacy will be positively associated with psychological wellbeing as measured by mental wellbeing and life satisfaction.

### Hypothesis 2

Seasonal variation in psychological wellbeing will be observed, with significantly higher mental wellbeing and life satisfaction in summer compared to autumn/winter.

### Hypothesis 3

*[if hypotheses 1 and 2 hold true]*: Digital self-efficacy will have a protective influence on the observed seasonal related reductions in psychological wellbeing (i.e. mental wellbeing and life satisfaction).

## Methods

### Overview of study design

An exploratory survey design was used, with the survey repeated four times during a year: July 2021, November 2021, March 2022 and July 2022. As the majority of questionnaires were completed online, the CHERRIES Checklist for Reporting Results of Internet E-Surveys [34] was used to guide the survey design, conduct and reporting. Ethical approval was obtained as part of the wider Smartline project, approved by the University of Exeter Business School Ethics Committee (Ref: eUEBS002996, 10/12/2019).

### Recruitment and data collection

All current participants in the Smartline project (n = 167 in July 2021) were invited to take part in the survey. The Smartline cohort comprises social housing residents aged 18 + living in properties managed by Coastline Housing in West Cornwall. The invitation to participate in the survey was addressed to the tenancy holder (i.e. one person per household). To minimise digital exclusion in the research process, both online and postal questionnaires were used. For participants with e-mail addresses, an information sheet (Supporting File 1) with a link to the first questionnaire was sent via e-mail in July 2021. Participants without e-mail addresses were sent the same questionnaire via post, together with the information sheet and a Freepost envelope addressed to the research office. The same procedure was used for the three subsequent questionnaires. For accessibility reasons, all participants were given the option to request a postal questionnaire. Respondents received incentives for participation – a £10 shopping voucher after completing two questionnaires, and a further £10 voucher on completion of all four questionnaires. All online questionnaires were completed via the Happiness Pulse online survey platform [35]; postal responses were inputted to this platform by the research team. Participants receiving the questionnaire in digital format were sent two e-mail reminders (after five and ten days), and postal questionnaire recipients had a follow-up telephone call from the research team if they had not completed the questionnaire within 14 days.

### Questionnaire content

The questionnaire consisted of the core Happiness Pulse wellbeing questions [36] and a bespoke digital module that was developed by the Smartline team as part of the wider project. The questionnaire was designed to assess the wellbeing and digital engagement of the Smartline participants throughout the project. For the seasonal survey, the same questionnaire was used for all four timepoints.

The Happiness Pulse is a survey instrument with known validity and reliability that uses self-rated Likert scales to assess four domains of psychosocial wellbeing [37]. The domains are general wellbeing, emotional or mental wellbeing, behavioural wellbeing, and social wellbeing.

The digital module used similar self-rated Likert scale items to assess attitudes to technology (e.g. perceived usefulness), behaviours (e.g. frequency of use) and digital competence (e.g. self-efficacy). Questions on specific technologies (video calls and messaging) and technology in general were included. The digital module was informed by theories of behaviour change [38, 39] and technology acceptance [40–42]. The module included a combination of theory-based questions created by the research team and questions adapted from existing sources including the UK Government Digital Inclusion Evaluation Toolkit [43, 44]. A demographics section captured information on participants’ gender, age, racial and cultural identity, and whether they considered themselves to have a disability. Participants provided a unique identifier (initials and date of birth) to enable tracking of responses and removal of duplicates. The questionnaire containing the Happiness Pulse and digital module is included in Supporting File 2. Providing a unique insight into the wellbeing of social housing residents throughout a year, the complete survey data may be used in future studies and is available to other researchers on request.

### Selection of variables for analysis

#### Psychological wellbeing

The psychological wellbeing variables selected for this study were:

- The short Warwick-Edinburgh Mental Wellbeing Scale (SWEMWBS) mental wellbeing summary score. This was a single score (responses on a scale from 7 to 35) comprised of seven individual items: optimism, worth, peace of mind, resilience, competence, autonomy and relationships [45–47].
- The ONS-4 life satisfaction question [48], a measure of general wellbeing within the Happiness Pulse [36]: “Overall, how satisfied are you with your life nowadays?” (responses measured on a 0–10 scale from ‘not at all’ to ‘completely’).

These two variables were selected as they are widely used and well validated measures of psychological wellbeing (the behavioural and social wellbeing questions focused more on behaviours such as frequency of exercise and socialising).

A time variable was used to assess seasonal changes, with each completed questionnaire assigned a number from 1 (July 2021) to 4 (July 2022).

#### Digital competence

The general technology self-efficacy question (“I feel confident that I am able to use most types of digital technology to do the things that I want to do”, responses on a 1–5 Likert scale of ‘strongly disagree’ to ‘strongly agree’) was selected as the digital competence measure. This was deemed an encompassing measure of digital competence; self-efficacy has been recognised as the ultimate outcome of digital competence [8].

### Data analysis

One hundred and ten respondents participated in at least one survey, 30 respondents completed only one questionnaire, 21 completed two questionnaires, 29 completed three questionnaires, and 30 completed all four questionnaires. Twenty four people (approximately 22%) opted to complete paper questionnaires.

Simple descriptive statistics (i.e. frequencies and percentages) were calculated for demographic data and digital competency for the 110 participants that completed at least one survey. The SWEMWBS scoring protocol was followed to calculate a summary mental wellbeing score for each individual at each timepoint; the seven individual item scores were summed and then transformed according to national norms [49]. Means and standard deviations for the sample were then calculated for the psychological wellbeing outcomes.

Ordinary least squares (OLS) regression with robust standard errors was used to explore the associations between digital self-efficacy and mental wellbeing, and between digital self-efficacy and life satisfaction (Hypothesis 1). Key demographic variables were controlled for in the models (age, gender and disability). This analysis used data from the first completed questionnaire for each respondent.

Repeated measures analysis of variance (ANOVA) was used to explore seasonal changes in mental wellbeing and life satisfaction (Hypothesis 2). ANOVAs were performed using two subsets of the data – 30 respondents completing all four questionnaires (to test changes between all four waves of the survey) and 59 respondents completing the November 2021 and July 2022 questionnaires (to provide an autumn/winter and summer comparison). To examine potential changes in wellbeing while controlling for age, gender and disability, an OLS panel analysis was estimated using data for the 59 participants that completed the November 2021 and July 2022 questionnaires.

For hypothesis 3, we intended to test for interactions between the digital self-efficacy and time variables. However, this was not feasible due to the lack of significant results for hypothesis 2 and insufficient sample size.

The data were organised in Excel [50] and all analysis was performed in Stata version 17 [51].

## Results

### Respondent characteristics including digital self-efficacy

The characteristics of the 110 respondents are summarised in Table 1. Respondents were older, with 45% aged 65 + years, two thirds were female, and a high proportion (59%) reported a disability. The majority (94%) were white, and 90% identified as British, British Cornish or Cornish. Digital competence levels varied greatly. Regarding general technology self-efficacy, 64% of respondents felt confident that they were able to use digital technology to do the things they wanted to do.

### Psychological wellbeing: mental wellbeing and life satisfaction

Psychological wellbeing scores were wide ranging (Table 2). The mean mental wellbeing (SWEMWBS) scores were 23.4 ± 4.8 for males and 22.6 ± 4.0 for females in our sample; this is slightly (although not significantly) lower than the national (England) male and female norm scores of 23.7 and 23.2 respectively [46]. The mean life satisfaction score was 6.8 ± 2.4, compared to the UK average of 7.4 [52].

**Table 1:** Participant demographics and digital competence (n = 110^a^)

Variable	Respondents n (%^b^)
Age (years)	
25–34	7 (6)
35–64	54 (49)
65±	49 (45)
**Gender**	
Male	35 (32)
Female	73 (66)
Non-binary	1 (1)
Prefer not to say	1 (1)
**Disability**	
Yes	65 (59)
No	39 (35)
Prefer not to say	6 (5)
**Racial identity**	
White	103 (94)
Mixed race	2 (2)
Other	2 (2)
Prefer not to say	3 (3)
**Cultural identity**	
British	41 (37)
British Cornish	31 (28)
Cornish	27 (25)
Scottish/Irish/Welsh	3 (3)
Other	4 (4)
Prefer not to say	4 (4)
**Digital self-efficacy** *“I feel confident that I am able to use most types of digital technology to do the things that I want to do”*	
1 = Strongly disagree	8 (7)
2 = Disagree	15 (14)
3 = Neither agree nor disagree	17 (15)
4 = Agree	45 (41)
5 = Strongly agree	25 (23)

^a^ Table includes data from the first completed questionnaire for each respondent.

^b^ Percentages may not total 100 due to rounding.

**Table 2:** Psychological wellbeing scores overall and by gender (n = 110^a^)

Variable	Mean (standard deviation)			Range of scores (potential range)
	**Overall** ^b^ **(n = 110)**	**Male** **(n = 35)**	**Female** **(n = 73)**	
Mental wellbeing (SWEMWBS)	22.7 (4.4)	23.4 (4.8)	22.6 (4.0)	11.3–32.6 (7.0–35.0)
Life satisfaction	6.8 (2.4)	6.8 (2.3)	6.9 (2.3)	0–10 (0–10)

^a^ Table includes data from the first completed questionnaire for each respondent.

^b^ Scores for ‘other’ and ‘prefer not to say’ gender not reported to protect confidentiality.

#### Hypothesis 1

Associations between digital self-efficacy and psychological wellbeing.

Two OLS regression models were run to examine the association between digital self-efficacy and (a) mental wellbeing and (b) life satisfaction. Here we found that people with higher digital self-efficacy reported higher mental wellbeing as assessed by the SWEMWBS score and this relationship was statically significant (p = 0.001) when controlling for age, gender and disability status (Table 3). Similarly, controlling for age, gender and disability status, people with higher digital self-efficacy reported greater life satisfaction (p = 0.002).

Of note, the older age group in this sample (65 + years) had significantly higher mental wellbeing than those aged 35 to 64 years (p < 0.001). People reporting one or more disabilities had significantly lower life satisfaction than those with no disabilities (p = 0.006).

**Table 3:** OLS regression of the relationship between digital self-efficacy and (a) mental wellbeing and (b) life satisfaction controlling for age, gender and disability (n = 110)

	(a) Mental wellbeing	(b) Life satisfaction
digital self-efficacy	1.305^***^ (0.001)	0.555^***^ (0.002)
age25_34 ^a^	1.295 (0.462)	-0.442 (0.567)
age65 ^a^	3.391^***^ (< 0.001)	0.798^*^ (0.084)
female ^b^	-0.155 (0.859)	0.336 (0.474)
disability ^c^	-1.040 (0.191)	-1.213^***^ (0.006)
R-sqr	0.200	0.147
Number of respondents	110	110

^*^p < 0.10, ^**^p < 0.05, ^***^p < 0.01; p-value in parentheses.

^a^ Ref: age 35 to 64 ^b^ Ref: male ^c^ Ref: no disability

#### Hypothesis 2

Seasonal changes in psychological wellbeing.

The mean mental wellbeing and life satisfaction scores by season are presented in Table 4.

**Table 4:** Psychological wellbeing (mental wellbeing and life satisfaction) by season

a) Respondents with autumn/winter and summer data (n = 59)				
	**Mean (standard deviation)**			
	**Nov-21**		**Jul-22**	
Mental wellbeing (SWEMWBS)	23.4 (4.6)		23.2 (4.4)	
Life satisfaction	7.5 (2.1)		7.3 (2.3)	
b) Respondents with complete data for all four surveys (n = 30)				
	**Mean (standard deviation)**			
	**Jul-21**	**Nov-21**	**Mar-22**	**Jul-22**
Mental wellbeing (SWEMWBS)	23.4 (4.4)	24.0 (4.6)	23.9 (4.5)	23.5 (4.4)
Life satisfaction	7.5 (2.0)	7.8 (1.9)	7.6 (1.9)	7.4 (1.9)

The ANOVA analysis found no significant changes in mental wellbeing between all four waves of the survey (*F* = 1.06, p = 0.372) or between autumn/winter and summer (*F* = 0.54, p = 0.464). Similarly, there were no significant changes in life satisfaction between all four survey waves (*F* = 1.06, p = 0.372) or between autumn/winter and summer (*F* = 2.1, p = 0.152). The panel analysis controlling for age, gender and disability (Table 5) confirmed these findings; mental wellbeing and life satisfaction did not significantly change between autumn/winter and summer (p > 0.1). The ANOVA results also showed that there were no significant changes in digital self-efficacy between all four surveys waves (*F* = 1.13, p = 0.341) or between autumn/winter and summer (*F* = 1.25, p = 0.295).

**Table 5:** Panel analysis of seasonal changes in mental well-being and life satisfaction (n = 59)

	Mental wellbeing (SWEMWBS)	Life satisfaction
summer ^a^	-0.251 (0.486)	-0.260 (0.182)
age25_34 ^b^	3.066^***^ (0.003)	0.471 (0.527)
age65 ^b^	4.047^***^(< 0.001)	1.151^**^ (0.024)
female ^c^	-2.143^*^ (0.080)	-0.857^*^ (0.081)
disability ^d^	-1.312 (0.183)	-0.788^*^ (0.092)

^*^p < 0.10, ^**^p < 0.05, ^***^p < 0.01; p-value in parentheses.

^a^ Ref: winter ^b^ Ref: age 35 to 64 ^c^ Ref: male ^d^ Ref: no disability

#### Hypothesis 3

Digital self-efficacy as a potential protective factor for seasonal changes in psychological wellbeing.

As no seasonal variation in psychological wellbeing was observed (Hypothesis 2), and sample size was too small to test for interactions in the panel analysis, the role of digital self-efficacy in maintaining wellbeing through the year was not explored.

## Discussion

This is the first study to explore associations between digital competence (assessed by general technology self-efficacy) and psychological wellbeing, in addition to considering seasonal changes in wellbeing for social housing residents. We found a significant positive association between digital self-efficacy and mental wellbeing, and between digital self-efficacy and life satisfaction (Hypothesis 1). Extending previous findings beyond student and elderly populations, these findings have important public health implications and suggest a need to improve digital competence in social housing residents as a potential pathway to higher wellbeing. Increasing digital skills and engagement leads to a number of benefits, including greater opportunities for work, education and socialising, and providing access to resources for managing finances and health [53]. Our findings suggest that improving digital competence is also likely to benefit psychological wellbeing. Consequently, benefits from a healthcare perspective are expected; research has found that good mental wellbeing lowers the risk of the onset of several chronic diseases including cardiovascular diseases [54], increases life expectancy [55] and is associated with healthy aging more broadly [56].

Regarding seasonal variations in wellbeing, there was no evidence of seasonal changes in either mental wellbeing or life satisfaction in the survey respondents (Hypothesis 2). Given the lack of observed seasonal variation in wellbeing and small sample size, we did not explore the role of digital competence as a protective factor against seasonal related reductions in psychological wellbeing (Hypothesis 3). However, these hypotheses should be tested with a larger sample as there is significant evidence of seasonal changes in mental wellbeing in the general adult population [13, 15], and groups such as social housing residents may be more vulnerable to the adverse psychological impacts of factors such as fuel poverty which are most commonly experienced in winter [20, 21].

As noted, psychological wellbeing is a complex phenomenon that is influenced by many different individual, community, and place-based factors [17]. Whilst this study indicates that there is an association between psychological wellbeing and digital self-efficacy, there is a need for further research on the mechanisms through which digital self-efficacy and digital competence more broadly lead to improved wellbeing. Although this was beyond the scope and scale of this study, the literature on technology use and wellbeing suggests various possible mechanisms. A large proportion of existing research has focused on the benefits of communications technologies for social wellbeing, including helping to increase social connections and reduce loneliness [2, 4, 5, 57]. For example, older people who more frequently use digital devices report lower depressive symptoms and the benefits are greater for those with limited social contacts [5]. Other potential pathways which have been less studied include improved access to online health information and resources [2] and improved health literacy [3]. Technology provides increased opportunities for professional development [6] and enables the development of new skills which increases general self-efficacy [7]; these are also likely to contribute to improved wellbeing. It is conceivable that being competent with technology increases resilience and provides people with the resources to cope with problems. The possibility of reverse causation should also be considered; for example, people with higher psychological wellbeing may be more likely to engage in behaviours that lead to greater digital self-efficacy and digital competence, such as digital skills training. Both pathways could operate together leading to a positive feedback loop of improved wellbeing, digital self-efficacy and digital competence.

Although the survey respondents included a range of individuals with varying levels of digital self-efficacy and psychological wellbeing, the sample comprised of a large proportion of older adults from a single social housing provider; this limits generalisability. The findings may differ in social housing residents in other regions nationally and internationally. Larger scale studies with mediation analysis and qualitative studies in diverse populations will help to elucidate the mechanisms and improve our understanding of the complex pathways involved. Research should seek to determine which specific digital competencies are related to different aspects of wellbeing. A better understanding of the mechanisms in different populations and contexts will facilitate the development of tailored interventions.

Future studies should use objective assessments of digital competence where possible. This is a recognised limitation of the present study, but it was deemed necessary to have a single, encompassing measure of digital self-efficacy that could be easily understood and completed by all the respondents, and that could be used in both online and paper questionnaires. The Happiness Pulse and digital module had been successfully used to assess wellbeing and digital competence in forthcoming Smartline studies (pending publication), and was selected for reasons of familiarity to participants, consistency and comparability.

Although this was a small-scale study, the survey tool and methods may be used as a blueprint for larger population surveys. The questionnaire could be used or adapted by social housing organisations or councils to explore digital competence and wellbeing of residents, and to inform the development and evaluation of interventions. The repeated survey approach is recommended to explore seasonal changes and provide stronger evidence of causal links in the relationships between technology and wellbeing [4, 12].

Social housing residents are at high risk of both digital and social exclusion [30, 58]. In our previous qualitative work that explored barriers to technology use in this group, privacy, safety and security were major concerns, together with a fear of ‘getting it wrong’ [59]. While improving digital skills, literacy, and access are vital, there is a need to improve additional aspects of digital competence, including attitudes towards and general confidence in using technology as a pathway to improve mental wellbeing and life satisfaction in social housing residents.

## Conclusions

In this repeated survey study of social housing residents, we found evidence of a positive association between digital self-efficacy and mental wellbeing, and between digital self-efficacy and life satisfaction. The findings extend the existing literature beyond student and elderly populations and suggest that improving digital competence is a potential pathway to improving wellbeing in social housing residents. Surveys with larger samples and qualitative studies are needed to establish whether the findings may apply to other socio-economically disadvantaged populations, identify any seasonal effects, and elucidate the mechanisms involved. Our findings highlight the value of investing in digital inclusion for social housing organisations, public health practitioners, councils, commissioners, and policymakers, and may also be of interest to technology developers and marketers.

### Electronic supplementary material

Below is the link to the electronic supplementary material.

Supporting File 1: Participant information sheet

Supporting File 2: The questionnaire including the Happiness Pulse and digital module

## Data Availability

The data that support the findings of this study are stored in the University of Exeter ORE (Open Research Exeter) repository (10.24378/exe.4444) and are available in an anonymised form upon reasonable request (access requests must be made through the online platform).
