# Supplementary material for: Digital competence and psychological wellbeing in a social housing community: a repeated survey study
Source: BMC Public Health. 2023 Oct 13;23:2002. doi: 10.1186/s12889-023-16875-2 (PMC10576269; doi:10.1186/s12889-023-16875-2)
Supplement: Supplementary file 1 — Supporting File 1: Participant information sheet [file 12889_2023_16875_MOESM1_ESM.pdf]

SEASONAL WELLBEING SURVEY PROJECT INFORMATION SHEET FOR PARTICIPANTS

Thank you for your interest in this project.

Please read this information sheet carefully before deciding whether or not to take part.

What is Smartline?

- Smartline is a research project in partnership with the University of Exeter, Coastline Housing, Cornwall Council, Volunteer Cornwall and the South West Academic Health Science Network.
- Smartline is part funded by the European Regional Development Fund and Cornwall Council.
- Smartline is investigating how digital technology could help people live longer and healthier lives in stronger communities.
- Information about the Smartline project can be found online here: www.smartline.org.uk or if you would like to speak to us you can call Karen on **07968 706114** or Adrian on **01209 200200**.
- Smartline is launching a project to gain an understanding of your personal wellbeing and to start to think about how we can maintain and/or improve it as part of our work.

What is the Seasonal Wellbeing Survey Project?

- The Season Wellbeing Survey Project seeks to gain an understanding of your personal wellbeing over the course of a year from July 2021 to July 2022.
- To measure your wellbeing, the project will use the Happiness Pulse, a simple 20 minute survey. The Happiness Pulse measures an individual's wellbeing and gets to the heart of the how people feel and function in their lives, work and communities.

Who can participate?

This study is open to Smartline participants over the age of 18.

What does participation in this study involve?

- You will be asked to complete the Happiness Pulse four times over a period of a year: July 2021, November 2021, March 2022 and July 2022.
- The survey can be completed online or via a paper copy. If you complete online you will be given a wellbeing score at the end of the questionnaire.
- You may also be asked if you would like to be interviewed about your experiences. Interviews will take place at a time that is convenient for you, by telephone. We will audio-record the interview with your permission (the recording will only be accessed by the research team for note-taking purposes).

What will the surveys ask?

The survey will ask about your physical, emotional, and social wellbeing.

How long will the surveys take?

The survey takes approximately 20 minutes.

What if I change my mind about participation in the study?

You can withdraw from the study at any time by contacting the research team on **smartlineresearch@exeter.ac.uk** or Karen on **07968 706114** or Adrian on **01209 200200.**

Is there an incentive to participate?

Incentives are offered to all eligible Smartline participants. Upon completion of two surveys you will receive a £10 shopping voucher and those who complete all four will receive a further £10 shopping voucher.

How will my information be handled?

The University of Exeter processes personal data for the purposes of carrying out research in the public interest. The University will endeavour to be transparent about its processing of your personal data and this information sheet should provide a clear explanation of this. If you do have any queries about the University's processing of your personal data that cannot be resolved by the research team, further information may be obtained from the University's Data Protection Officer by emailing **dataprotection@exeter.ac.uk** or at **www.exeter.ac.uk/dataprotection.**

We will not collect or store any data without your consent. If you provide your consent your data will be handled in the following ways:

- After completing the wellbeing survey, your information will be anonymised and processed and coded with study numbers so no names will be retained.
- The anonymised and processed data will be stored in a repository and kept until 31 December 2033 as required by the project funder.
- Only the project team will have access to a list containing identifiable information such as your name, address, phone number, study number, so we can contact you during the study if needed. This list will be securely stored on the University server with access limited to the core project team.
- Any printed copies of the anonymised survey will be stored in a locked cupboard at University of Exeter Medical School, Knowledge Spa.
- This information will also be kept until 31 December 2033 as required by the project funder. Your personal information will not be shared with any third party. You have a right to access your own data or for it to be deleted.

If you have any concerns regarding the handling of your personal, please contact the research team on **smartlineresearch@exeter.ac.uk** or Karen on **07968 706114** or Adrian on **01209 200200.**

Who should I contact if I have any further questions?

If you have any questions about our project, either now or in the future, please feel free to contact:

Smartline research team.
Email: **smartlineresearch@exeter.ac.uk**

Karen Spooner
Smartline Community Engagement Officer, Volunteer Cornwall
Telephone: **07968 706114**

Adrian Ankers
Smartline Project Co-ordinator, Coastline Housing
Telephone: **01209 200200**

Smartline has been reviewed and approved by the University of Exeter Business School Ethics Committee

Reference number: eUEBS002996 Date: 10/12/2019
